# Draft Genome Sequences of *Saccharopolyspora* sp. Strains and *Streptomyces* sp. Strains, Isolated from Social Wasps (Vespidae; Polistinae: Epiponini)

**DOI:** 10.1128/MRA.00935-21

**Published:** 2022-01-06

**Authors:** Mariela Gutiérrez-Araya, Kattia Núñez-Montero, Javier Pizarro-Cerdá, Laura Chavarría-Pizarro

**Affiliations:** a Centro de Investigación en Biotecnología, Instituto Tecnológico de Costa Rica, Cartago, Costa Rica; b Laboratorio de Biología Molecular Aplicada, Centro de Excelencia en Medicina Traslacional, Universidad de La Frontera, Temuco, Chile; c Institut Pasteur, Université de Paris, Yersinia Research Unit, Paris, France; Indiana University, Bloomington

## Abstract

Strains of the genera *Saccharopolyspora* and *Streptomyces* were isolated from *Protopolybia* sp. and *Metapolybia* sp. social wasps in Costa Rica. Draft genome sequences were obtained for six isolates, ranging from 6.4 Mb to 9.1 Mb long and having GC contents of 71 to 73%.

## ANNOUNCEMENT

Underexplored sources of natural products have recaptured the interest of scientists, and these include new bacterial strains from social insects as promising sources of novel molecules (1). Interest in these microbes is due to their ability to adapt and compete with a broad spectrum of insect pathogens, which has allowed their evolutionary success over the time (2) and might provide them with an untapped diversity of metabolites. Under this premise, *Saccharopolyspora* sp. and *Streptomyces* sp. symbiotic strains associated with social wasps from the genera *Metapolybia* and *Protopolybia* were isolated.

Actinobacteria strains were isolated from the cuticle and salivary glands of social wasps collected at three different locations in Costa Rica (Table 1). For bacterial isolation from the cuticle, one worker wasp was rubbed directly onto a petri dish using sterile forceps. For actinobacterial isolation from the salivary glands, thoracic glands were homogenized in 30 μl PBS + 100 mM sodium acetate (pH 5), and 20 μl was plated. Three different media supplemented with nystatin (0.1%) and nalidixic acid (10 μg/ml) were used for isolation: 1% Luria-Bertani (LB; Miller) medium and International Streptomyces Project 1 and 2 (ISP1 and ISP2) media. The plates were incubated at room temperature (∼22°C) for 1 month; colonies exhibiting macroscopic morphologies of actinobacteria were transferred and purified onto new LB, ISP1, or ISP2 plates (Table 1). Genomic DNA from six pure isolates was obtained by phenol-chloroform extraction as described by Chun and Goodfellow (3), using 1 mg of bacterial colonies, aerial mycelia, and spores of each strain after 7 days’ culture at room temperature on their respective media. Genomic libraries were prepared using the Nextera XT DNA library preparation kit (Illumina, San Diego, CA) and sequenced using a paired-end strategy of 2 × 150 nucleotides on the Illumina NextSeq 500 platform. The raw sequencing reads were both quality controlled and trimmed using fqCleanER (https://gitlab.pasteur.fr/GIPhy/fqCleanER) with all the “-s” options activated and clipping of the Illumina Nextera index. *De novo* assemblies were obtained from trimmed reads using SPAdes v3.14.1 (4) with the following options: -k 21,33,55,77 --only-assembler --careful. The quality of the assembled genomes was verified using CheckM v1.1.3 (5). Default parameters were used for data processing except where otherwise noted.

**TABLE 1:** Characteristics of six *Actinobacteria* isolates associated with social wasps and sequencing general results

Metric	Data for strain:^*a*^					
	*Saccharopolyspora* sp. 6M	*Saccharopolyspora* sp. 6T	*Saccharopolyspora* sp. 6V	*Saccharopolyspora* sp. 7B	*Streptomyces* sp. 7G	*Streptomyces* sp. 8L
Source (individual ID)	Cuticle of *Metapolybia* sp. (insect 1)	Cuticle of *Metapolybia* sp. (insect 1)	Cuticle of *Metapolybia* sp. (insect 2)	Cuticle of *Metapolybia* sp. (insect 3)	Salivary gland of *Protopolybia* sp. (insect 4)	Cuticle of *Protopolybia* sp. (insect 5)
Sampling date (yr-mo-day)	2019-6-20	2019-6-20	2019-9-19	2019-9-19	2019-8-16	2020-2-12
Sampling location	Santa Cruz, Guanacaste (10°16′22.8″N, 85°31′43.5″W)	Santa Cruz, Guanacaste (10°16′22.8″N, 85°31′43.5″W)	Santa Cruz, Guanacaste (10°16′22.8″N, 85°31′43.5″W)	Santa Cruz, Guanacaste (10°16′22.8″N, 85°31′43.5″W)	Research Station La Selva, Heredia (10°25′19″N, 84°00′54″W)	Golfito, Puntarenas (8°39′15.8″N, 83°10′45.1″W)
Isolation and culture medium	LB	LB	ISP1	ISP2	ISP1	ISP2
Total no. of reads	4,447,755	4,414,784	5,429,465	4,959,684	5,077,834	6,368,654
Genome size (bp)	6,438,697	6,587,366	6,571,055	6,480,769	7,848,592	9,078,753
No. of genes annotated with PGAP (7)	5,764	5,898	5,942	5,725	7,357	8,045
No. of RNAs annotated with PGAP (7)	54	56	56	55	73	78
% GC	73	72	72	73	71	71
No. of contigs	360	355	242	291	1,081	991
*N*_50_ (bp)	29,651	38,839	50,652	40,461	12,009	15,698
Completeness (%)	99.65	99.47	99.47	99.82	99.33	98.19
Contamination (%)	1.48	1.45	1.12	1.45	1.99	2.17
Sequencing depth (×)	100	98	112	107	99	103
BioSample accession no.	SAMN21501660	SAMN21501661	SAMN21501662	SAMN21501663	SAMN21501664	SAMN21501665
GenBank WGS^*b*^ accession no.	JAIUJZ000000000	JAIUKA000000000	JAIUKB000000000	JAIUKC000000000	JAIUKD000000000	JAIUKE000000000
GenBank assembly accession no.	GCA_020176775.1	GCA_020177455.1	GCA_020176785.1	GCA_020177435.1	GCA_020177475.1	GCA_020176795.1
SRA accession no. for raw reads	SRX13158144	SRX13158145	SRX13158146	SRX13158147	SRX13158148	SRX13158149

a Strain identification was based on ANI comparison with available species of the closest genus.

b WGS, whole-genome sequencing.

Six draft genome sequences were obtained, from 6.4 Mb to 9.1 Mb long. The relevant genome characteristics are presented in Table 1. A taxonomic comparison made using JSpeciesWS (6) based on average nucleotide identity (ANI) showed four *Saccharopolyspora* sp. strains sharing >99% genome identity among them and two different *Streptomyces* sp. strains. However, none of the comparisons showed results above the species threshold (>95%) with available genomes of the genera, suggesting that these isolates might be new species.

### Data availability.

The data from this study are available under BioProject accession number PRJNA764377. The BioSample material, assembled genomes, and raw reads have been deposited at DDBJ/ENA/GenBank under the accession numbers listed in Table 1.
